# Preoperative denosumab treatment in patients with giant cell bone tumors in limbs: A retrospective study using propensity score matching

**DOI:** 10.1002/cam4.5870

**Published:** 2023-05-22

**Authors:** Yongfu Huang, Mingxian Xu, Bo Wang, Zhiqiang Zhao, Tiao Lin, Gang Huang, Junqiang Yin, Xianbiao Xie, Jingnan Shen, Changye Zou

**Affiliations:** ^1^ Department of Orthopedic Oncology The First Affiliated Hospital of Sun Yat‐sen University Guangzhou Guangdong China

**Keywords:** denosumab, joint preservation, propensity‐score matching, surgical downgrading

## Abstract

**Background and Objectives:**

Denosumab is recommended for advanced giant cell tumor of bone (GCTB) that is unresectable or resectable with unacceptable morbidity. But the effect of preoperative denosumab treatment on the local control GCTB remains controversial.

**Methods:**

We conducted a study of 49 patients with GCTB in the limbs treated with denosumab before surgery and 125 patients without in our hospital from 2010 to 2017. Propensity‐score matching (PSM) at a 1:1 ratio between the denosumab and control groups was performed to minimize possible selection bias, and compared the recurrence rate, limb function, and surgical degradation between the two groups.

**Results:**

The 3‐year recurrence rates in the denosumab group and the control group were 20.4% and 22.9% after PSM, respectively (*p =* 0.702). In the denosumab group, 75.5% (*n* = 37/49) of patients experienced surgical downgrading. Limb joint preservation rates were 92.1% (35) for 38 patients treated with denosumab and 60.2% (71) for 118 control subjects. (*p* ≺ 0.001). Postoperative MSTS were higher in patients in the denosumab group than in the control group (24.1 vs. 22.6, *p* = 0.034).

**Conclusions:**

Preoperative denosumab treatment did not result in an increased risk of local recurrence of GCTB. Patients with advanced GCTB may benefit from preoperative denosumab treatment for surgical downgrading and the preservation of the joint.

## Graphical abstract

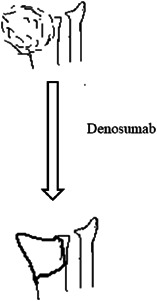

## 1 BACKGROUND

Giant cell tumor of bone (GCTB) is an intermediate locally aggressive primary bone tumor that commonly afflicts young adults within the metaphysis of the long bone (most common in the distal femur, proximal tibia, and distal radius), accounts for approximately 5% of all primary bone tumors, and occurs most frequently in individuals aged 20–40 years. En block excision and intralesional curettage are the two surgical treatment options for patients with resectable GCTB.^1^, ^2^, ^3^ However, local recurrence remains high (10%–50%).^4^ The recurrence rate after curettage is higher than after en bloc resection.^5^, ^6^ En bloc resection results in greater functional sacrifice in most cases, such as joint replacement. Currently, a reduction in the local recurrence of tumors and greater preservation of function are challenges faced in the treatment of GCTB.

Giant cell tumors are composed of osteoclast‐like giant cells and stromal cells. Neoplastic cells overexpress receptor activator of nuclear factor kappa‐B ligand (RANKL) and promote the recruitment of numerous reactive multinucleated osteoclast‐like giant cells, causing lacunar bone resorption. Denosumab, a human monoclonal antibody that inhibits RANKL, is approved for the treatment of adults and skeletally mature adolescents with GCTB that is unresectable or when surgical resection is likely to result in severe morbidity.^7^, ^8^ Reductions in the tumor size and calcification of tumors have been observed after denosumab treatment, which may reduce blood loss and facilitate tumor resection and downgrade the operation.^9^ Controversy exists regarding whether denosumab might affect recurrence of GCTB. Therefore, we retrospectively studied patients who were treated in our department in terms of both local recurrence and postoperative function to further discover the role of preoperative denosumab treatment.

## 2 MATERIALS AND METHODS

The study was approved by the Ethics Committee of the First Affiliated Hospital of Sun Yat‐sen University. We retrospectively reviewed the medical records of limb GCTB patients diagnosed with histologically confirmed GCTB in limb who were surgically treated in our department from 2010 to 2017. All patients were confirmed by preoperative biopsy and postoperative histopathology as GCTB. Follow‐up information of patients was obtained by telephone and outpatient service. Patients were surveyed every 3 months within 2 years, once every 6 months between 2 and 5 years, and once a year after 5 years using x‐ray, CT, and MRI.

The initial diagnosis and assessment of the recurrence of GCTB were based on the clinical symptoms, radiology examination, and pathological examination. The surgical plan was determined at the first visit according to the tumor location, Campanacci radiographic classification, joint and bone destruction, and mechanical stability. Intralesional extensive curettage was performed for Campanacci Grade I and II GCTB, while marginal resection was considered for Campanacci Grade III GCTB or if giant cell tumors had large bone damage, articular surface damage exceeding 50%, or were located in non‐weight‐bearing bones, such as the distal radius and proximal fibula.^5^, ^10^, ^11^ Under this standard, if patients with GCTB undergoing preoperative denosumab treatment become eligible for curettage in patients with planned resection, or patients with planned curettage can choose long‐term treatment with denosumab to avoid surgery, we believe that preoperative denosumab treatment plays a role in surgical degradation.

Forty‐nine patients received denosumab (Xgeva; Amgen) before surgery (at a dose of 120 mg on days 1, 8, 15, and 29 and then 4 weekly by subcutaneous injection). These patients purchased denosumab in Hong Kong and returned to Guangzhou for treatment. No consensus has been reached on the preoperative treatment of denosumab. In this study, denosumab was used not only for patients who plan to undergo curettage, but also for patients who planned to undergo resection. In this study, 93.8% (*n* = 46/49) of patients in the study received 4–6 times of denosumab treatment. Due to individual differences in drug efficacy, one patient received three doses of treatment and two patients received eight doses of treatment. After 3–8 doses of treatment were administered, patients were re‐evaluated using radiology examinations, including CT/MRI. If subchondral bone could be preserved in patients scheduled for en bloc resection after denosumab treatment, then extensive curettage was performed instead of en bloc resection. The tumor mass extending outside the bone was marginally resected, and the tumor within the bone was removed with curettage. Big enough bone window was opened to decrease blind corners as far as possible, the tumor cavity was soaked with iodine tincture, and then electrosurgical inactivation was performed point by point, followed by high‐speed burr to remove the burnt tissue. This procedure was repeated twice, and then the cavity was rinsed with sterile water and filled with auto/allograft or cement and fixed with plate.

We matched patients with 1:1 propensity score matching (PSM) by R(4.1.2) using factors that may influence recurrence (Campanacci grading, sex, Pathological fracture, Surgical method, tumor size, tumor location, age, and history of recurrence) to reduce the influence of selection bias on local control of GCTB. Forty‐nine patients of each group were included in comparison study after PSM.

The Pearson's chi‐squared test or Fisher's exact test was used to assess changes in surgical downgrading after denosumab preoperative therapy. The Kaplan–Meier method was used to assess relapse‐free survival, and the log‐rank test was used to assess differences between survival curves. Cox regression analysis was performed to estimate the hazard ratio of risk factors for local recurrence and factors with a significant association in the univariate analysis (*p* < 0.1) were included in a multivariate analysis to identify independent risk factors for local recurrence. All analyses were performed using IBM SPSS (version 25.0, IBM).

## 3 RESULTS

One hundred seventy‐four patients with GCTB in the limbs were included in the current study, 49 patients in the denosumab group and 125 patients in the control group. Patients were followed up for an average of 32.1 months (7–66 months). Eighty‐one males (47%) and 93 females (53%) were analyzed. The median age was 30 (10–73) years. Fifty‐seven (32.8%) had tumors in distal femur, and 39 (22.4%) in proximal tibia, 27 (15.5%) in radius, 14 (8%) in proximal femur, 10 (5.7%) in humerus, 9 (5.2%) in distal tibia, 6(3.4%) in fibular head, 3 (1.7%) in distal ulna, and 9 (5.2%) in other sites. As shown in Table 1, 81 (46.6%) patients were diagnosed with Campanacci grade II tumors and 93 (53.4%) were diagnosed with grade III tumors. Forty‐three (24.7%) patients had a history of relapse and 31 (17.8%) patients were diagnosed with pathological fracture.

**TABLE 1:** Clinical characteristics of the 174 patients before propensity score matching.

	Denosumab group^a^	Control group	Sum	*p* Values
Mean age	29.9	33.2		0.073
Sex				0.949
Male	23	58	81	
Female	26	67	93	
History of recurrent				0.224
Yes	9	34	43	
No	40	91	131	
Pathological fracture				0.905
Yes	9	22	31	
No	40	103	143	
Campanacci				0.001*
II	13	68	81	
III	36	57	93	
Tumor location				0.042*
Radial, hand and foot	13	17	30	
Other bones	36	108	144	
Surgical method				<0.001*
Curettage	45	78	123	
Resection	4	47	51	
Mean tumor size(mm)	49.2	59.5		<0.001*

^a^ Preoperative denosumab was given subcutaneously at dosage of 120 mg for 3–8 times.

* *p*<0.05 indicated the distribution of patients was different between the two groups.

### 3.1 Local recurrence

**FIGURE 1:**
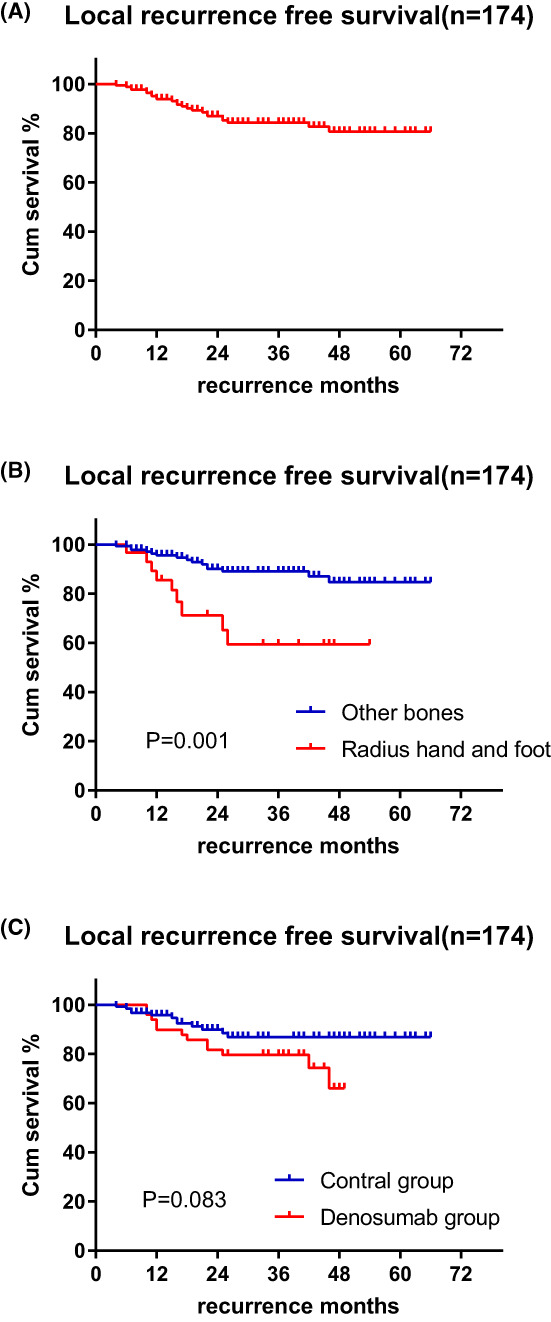
Kaplan–Meier estimated relapse‐free survival before PSM; (A) Overall relapse‐free survival; (B) Patients with GCTB in radius, hand and foot had lower relapse‐free survival; (C) Denosumab seems to result in lower relapse‐free survival.

Before PSM, 24 patients (*n* = 24/174) experienced local recurrence during follow‐up, and the 3‐year recurrence rate was 15.7% (Figure 1A). Univariate Cox regression analysis showed that younger people (*p* = 0.017), smaller tumor (*p* = 0.04) and tumors in the radial, hand and foot (*p* = 0.002) may correlated with a high recurrence rate. The 3‐year recurrence rate was 40.7% for patients with tumors located in the radius, hand and foot and 21% for patients with tumors located in other locations (*p* = 0.001) (Figure 1B). The 3‐year recurrence rate was 20.4% for patients receiving preoperative denosumab treatment and 13.1% for patients without preoperative denosumab treatment (*p* = 0.083) (Figure 1C). In the multivariate Cox regression analysis, no statistically significant differences in sex, pathological fracture, tumor size, history of recurrence, Campanacci staging, or surgical method, were observed (*p* > 0.05). Tumors in radial, hand and foot (*p* = 0.013) and younger patients (*p* = 0.018) were associated with higher recurrence rates. (Table 2).

**TABLE 2:** Univariate and multivariate Cox regression analysis before PSM.

Univariate Cox regression				Multivariate Cox regression		
Influence factor	HR	95% CIs	*p* Value	HR	95% CIs	*p* Value
Age	0.997	0.994 – 0.999	0.017*	0.996	0.994 – 0.999	0.018*
Denosumab neoadjuvant	2.005	0.897 – 4.481	0.09	1.265	0.537 – 2.982	0.591
History of recurrent	1.164	0.462 – 2.933	0.747			
Pathological fracture	1.112	0.415 – 2.980	0.833			
Tumor diameter	0.975	0.953 – 0.999	0.04*	0.997	0.970 – 1.025	0.834
Tumor in radius, hand and foot	3.756	1.636 – 8.625	0.002*	3.640	1.315 – 1.0074	0.013*
Female	1.055	0.473 – 2.356	0.896			
Campanacci III	1.033	0.458 – 2.331	0.938			

Abbreviations: CI, confidence interval; HR, hazard ratio.

* *p* < 0.05 indicated a strong correlation between the risk factor and recurrence

**FIGURE 2:**
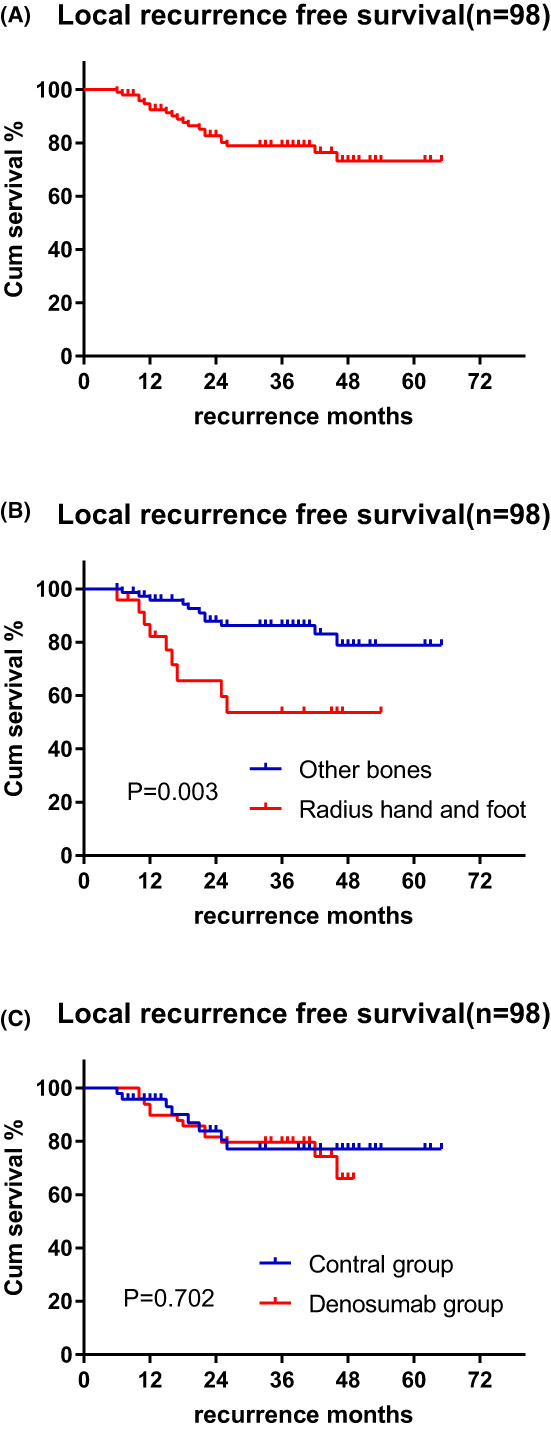
Kaplan–Meier estimated relapse‐free survival after PSM; (A) Overall relapse‐free survival (B) Patients with GCTB in radius, hand and foot had lower relapse‐free survival; (C) There was no significant difference in relapse‐free survival between the denosumab group and the control group.

When considering the composition of patients, we found more young, Campanacci III, and undergo curettage patients and radial site patients were in the denosumab group (Table 1). These factors had been reported to associate with higher risk of recurrence in previous and current study.^1^, ^12^ To reduce patient selection bias, we established 49 patients treated with denosumab as the baseline performed 1:1 PSM with factors that may influence local recurrence (Campanacci Grading, sex, Pathological fracture, Surgical method, tumor size, tumor location, age and history of recurrence). Finally, 49 patients without denosumab preoperative therapy were matched as the control group. The mean duration of follow‐up was 39.2 months in the denosumab group and 30.5 months in the control group. No significant differences were observed between the two groups in Campanacci Grade, tumor location, tumor size, and surgical method (Table 3). The 3‐year recurrence was 21.1% after PSM matching (Figure 2A), multivariate Cox regression analysis showed the tumor location was the only factor influencing local recurrence (*p* = 0.039) (Table 4). The three‐year recurrence rates were 46.4% and 13.7% for patients with GCTB in radius, hand and foot and other locations after PSM, respectively (Figure 2B). No significant difference in the recurrence rate was observed between the denosumab and control groups after PSM (20.4% vs. 22.9%, *p* = 0.702) (Figure 2C).

**TABLE 3:** Clinical characteristics of the 98 patients after propensity score matching.

	Denosumab group^a^	Control group	Sum	*p* values
Mean age	29.9	30.3		0.846
Sex				0.686
Male	23	25	48	
Female	26	24	50	
History of recurrent				0.460
Yes	9	12	21	
No	40	37	77	
Pathological fracture				
Yes	9	9	18	
No	40	40	80	
Campanacci				0.09
II	13	21	34	
III	36	28	64	
Tumor location				0.638
Radial, hand and foot	13	11	24	
Other bones	36	38	74	
Surgical method				
Curettage	45	43	88	0.505
Resection	4	6	10	
Mean tumor size	49.2	50.0		0.791

^a^ Preoperative denosumab was given subcutaneously at dosage of 120 mg for 3–8 times.

**TABLE 4:** Univariate and multivariate Cox regression analysis after PSM.

Influence factor	Univariate Cox regression			Multivariate Cox regression		
	HR	95% CIs	*p* Value	HR	95% CIs	*p* Value
Age	0.957	0.907 – 1.010	0.111			
Denosumab neoadjuvant	1.191	0.484 – 2.933	0.703			
History of recurrent	1.928	0.739 – 5.028	0.180			
Pathological fracture	1.536	0.558 – 4.227	0.406			
Tumor diameter	0.973	0.945 – 1.002	0.064	0.988	0.954 – 1.023	0.493
Tumor in radius, hand and foot	3.563	1.469 – 8.640	0.005*	2.945	1.054 – 8229	0.039*
Female	1.097	0.454 – 2.648	0.837			
Campanacci III	1.058	0.405 – 2.759	0.909			

Abbreviations: CI, confidence interval; HR, hazard ratio.

* *p* < 0.05 indicated a strong correlation between the risk factor and recurrence.

### 3.2 Surgery downgrading and function

In the denosumab group, five patients planned to undergo curettage, and 44 patients planned to undergo en bloc resection. After 3–8 doses of denosumab, 75.5% (*n* = 37/49) of patients who had planned to undergo resection ultimately opted for curettage instead (*p* < 0.001). Local recurrence occurred in 18.9% (*n* = 7/37) patients who underwent downgrading surgery and in 41.6% (*n* = 5/12) patients who did not (*p* = 0.111), there was no increased risk of recurrence associated with downgrading surgery after preoperative denosumab therapy. One patient underwent a second resection because of recurrence after curettage, finally 73.4% (*n* = 36/49) patient of the denosumab group underwent surgery downgrading.

**FIGURE 3:**
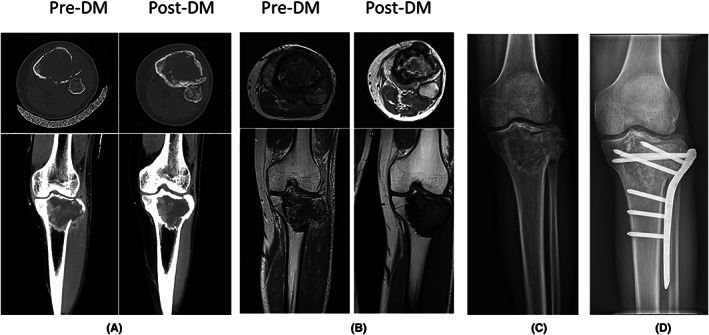
A 35‐year‐old men who was diagnosed with giant cell tumor (GCT) of proximal tibia and pathological fracture received preoperative denosumab treatment (six times). (A–C) CT, MRI, and x‐ray showed that the tumor was adjacent to the joint and involved the bone cortex. The peripheral sclerotic bone formation and the tumor margin became clear after denosumab treatment. (D) Curettage combined with internal fixation was performed, postoperative function was satisfactory without recurrence.

The joint preservation rate was 92.1% (35) for 38 patients in the denosumab group. Among which, six patients with pathological fracture performed curettage and kept the joint after preoperative denosumab treatment, as shown in Figure 3, while the joint preservation rate was 60.2% (71) for 118 control subjects (*p* < 0.001).

A VAS pain scale was used for the evaluation. After surgery, patients in the denosumab group had less pain than those in the control group (0.58 ± 0.839 vs. 1.82 ± 1.466, *p* < 0.001).

The average MSTS score of patients with limb tumors in the denosumab group was 24.1 ± 3.76, and the MSTS score of patients in the control group was 22.6 ± 3.95 (*p* = 0.034); patients in the denosumab group experienced better functional recovery after surgery among the patients surveyed.

## 4 DISCUSSION

The introduction of denosumab is a milestone in treatment of GCTB. Thomas et al reported an open‐label Phase II study with 86% (30 of 35 patients showing a tumor response to denosumab.^8^ Chawla et al. confirmed the efficacy (which included a reduction in the need for morbid surgery) and safety of denosumab in 282 patients with GCTB.^6^ The open‐label Phase II study conducted by Rutkowski et al assessed 222 patients with GCTB who were scheduled for surgical resection after treatment with denosumab. Of these patients, 48% (*n* = 106/222) received continuous denosumab treatment without surgery, 38% (*n* = 84) underwent surgery with less morbidity than planned, 24 underwent surgery as planned, and only 6 underwent surgery with greater morbidity than planned.^13^ The study shows that preoperative denosumab therapy resulted in beneficial surgical downgrading. Denosumab facilitates en bloc resection of GCTB because of a decrease in the vascular density, leading to less perioperative blood loss and shorter operating times.^14^ A study of 68 patients by C. Y. Lim et al showed that the recurrence‐free survival rate was significantly higher for the adjuvant denosumab group than for the group without adjuvant denosumab during the first 2 years postoperatively: 100% versus 83.8% at 1 year and 95.0% versus 70.3% at 2 years. However, no significant difference in the 3‐year recurrence‐free survival rate was observed.^15^ The authors proposed that preoperative denosumab treatment is beneficial for local control in the early postoperative period.

However, some studies suggested that preoperative denosumab treatment followed by curettage might lead to higher recurrence rates. Shinji Tsukamoto et al reported a local recurrence rate ranging from 20% to 100% in the curettage with preoperative denosumab group and 0%–50% in the curettage‐alone group. The authors concluded that the use of denosumab before curettage might be associated with a higher risk of recurrence.^16^ A recent meta‐analysis of 1082 patients (169 in the denosumab group and 913 in the control group) by Wenwei Qian et al. indicated that denosumab was associated with significantly higher risk of recurrence (*p* < 0.02) and inferior 5‐year recurrence‐free survival (*p* = 0.000).^17^ Traub et al performed a small sample prospective nonrandomized study and reported that during a median follow‐up of 30 months (ranging from 20 to 45 months), 3 of the 20 patients with GCTB who had preoperatively received denosumab relapsed, and the local control rates were similar to study of patients with GCTB who underwent curettage alone reported by Errani.^18^ Traub theorized that tumor cells can “hide” within the thickened cortex and subchondral bone, which might increase the risk of local recurrence.^19^ This finding may account for the high recurrence rate after the use of denosumab. Methods to delineate the true extent of the tumor and achieve complete tumor clarity after denosumab treatment are a concern.

This study is one of the largest cohort studies of GCTB in the limbs among which, 174 patients, including 49 patients with preoperative denosumab treatment were enrolled. In addition, all of the patients came from one center and the operation was performed by one surgical team. At the beginning, the result showed that preoperative treatment with denosumab seems to increase the risk of recurrence in GCTB, even though not significant. When we analyzed the composition of patients, we found that there were more young, Campanacci III, curettage and radial, hand and foot site patients in the denosumab group. These factors may contribute to the high recurrence rate in the denosumab group.^1^, ^20^, ^21^, ^22^ Patient selection bias is an unavoidable problem in retrospective studies; the bias such as the proportion of patients with Campanacci III and distal radial tumors can also be seen in some other studies.^18^, ^23^PSM has advantages of dimension reduction and study design separation.^24^ After PSM, Campanacci grade, surgical method, tumor size, and sites were balanced in both the groups, and the result indicated that the preoperative denosumab treatment did not increase recurrence rates.

In addition to recurrence, the function of patients with GCTB should not be ignored as most of the patients with GCTB are young and active patients. Although en‐block resection could reduce recurrence, the function loss and the complication after joint replacement will accompany the patients for life long. Subchondral bone sclerosis after preoperative treatment with denosumab make joint‐sparing procedure possible for some aggressive GCTBs or GCTBs with pathological fracture. Guido Scoccianti et al reported the result of 12 patients with more aggressive lesions performed curettage and cryotherapy rather than resection after preoperative denosumab treatment, five patients experienced local recurrence, 10 patients successfully preserved the joint at the end of follow‐up.^25^ In David's study, the joint salvage rate is 92% (23/25) in patients with high‐risk GCTBs treated with neoadjuvent denosumab and the recurrence rate was 44% after the median follow‐up of 57 months.^26^ In the current study, the joint salvage rate was 92.1% in the denosumab group which is higher than that in the control group. The denosumab group had less pain and better postoperative limb function; this was most possibly owing to higher joint salvage rate. Some patients may recurrence after curettage; more than 60% patients could perform repeated curettage and denosumab had also proved to be efficient and safety in long term use. Thus, the strategy of treatment for GCTBs with high risk recurrence should be reconsidered to balance both disease control and function.

## 5 CONCLUSION

Preoperative denosumab treatment of GCTB did not result in an increased risk of local recurrence when the residual tumor cell was treated properly, but it decreased the surgical grade and allowed the patient to retain better function after surgery. Therefore, we recommend preoperative denosumab treatment for patients with Campanacci grade III GCTB and patients requiring more aggressive surgery which may sacrifice the joint. To eliminate the residual tumor cells as far as possible, we can apply the extensive curettage combining penetrative heat inactivation and high‐speed burr. Using the electrocoagulation at spray model, the depth of heat inactivation in the bone can be more than 5 mm, which could inactivate the residual tumor cells hiding inside. As this study employed a retrospective design, more collaborative prospective randomized controlled studies should be conducted to clearly define the effect of preoperative denosumab treatment on the local control and functional recovery of patients with GCTB.

## AUTHOR CONTRIBUTIONS

**Yongfu Huang:** Conceptualization (equal); methodology (equal); writing – original draft (lead); writing – review and editing (equal). **Mingxian Xu:** Formal analysis (equal); software (lead); visualization (equal). **Bo Wang:** Data curation (equal); validation (equal). **Zhiqiang Zhao:** Investigation (lead). **Tiao Lin:** Data curation (equal). **Gang Huang:** Methodology (equal); supervision (equal). **Junqiang Yin:** Investigation (equal); supervision (equal). **Xian‐Biao Xie:** Investigation (equal); validation (equal). **Jingnan Shen:** Conceptualization (equal); supervision (supporting). **Changye Zou:** Conceptualization (supporting); funding acquisition (lead); writing – review and editing (equal).

## FUNDING INFORMATION

This work is supported by CSCO‐GCT research Project(Y‐2020GCT‐003) for follow up patients.

## CONFLICT OF INTEREST STATEMENT

The authors declare that they have no competing interests

## ETHICAL APPROVAL

The study was approved by the Ethics Committee of the First Affiliated Hospital of Sun Yat‐sen University.

## Data Availability

The data that support the findings of this study are available from corresponding author Changye Zou upon reasonable request.
